# Spontaneous pulmonary artery thrombus in a neonate

**DOI:** 10.1186/s43044-021-00167-4

**Published:** 2021-05-03

**Authors:** Y. S. Shrimanth, Krishna Prasad, Adari Appala Karthik, Parag Barwad, C. R. Pruthvi, Atit A. Gawalkar, Krishna Santosh, Sanjeev Naganur

**Affiliations:** 1 Department of Cardiology, Advanced Cardiac Centre, Post Graduate Institute for Medical Education and Research (PGIMER) Chandigarh, Sector 12, Chandigarh, 160 012 India; 2 Department of Pediatrics, Post Graduate Institute for Medical Education and Research (PGIMER) Chandigarh, Chandigarh, India; 3 Department of Cardiology, IMS BHU, Varanasi, India

**Keywords:** Pulmonary artery thrombus, Echocardiogram, Computed tomography pulmonary angiography, Persistent pulmonary artery hypertension

## Abstract

**Background:**

Pulmonary artery thrombosis is rare in neonates and mimics as persistent pulmonary hypertension or congenital heart disease. Risk factors include septicemia, dehydration, polycythemia, maternal diabetes, asphyxia, and inherited thrombophilias. They present with cyanosis and respiratory distress. Careful echocardiogram assessment helps in identifying the thrombus in the pulmonary artery and its branches. Computed tomography pulmonary angiography confirms the diagnosis.

**Case presentation:**

We present a case of term neonate who presented with respiratory distress and cyanosis and a detailed echocardiogram revealed thrombus in the origin of left pulmonary artery. The neonate was managed initially with unfractionated heparin and later with low molecular weight heparin with which there was significant resolution of the thrombus

**Conclusion:**

Spontaneous pulmonary artery thrombosis though rare should be suspected in any cyanotic neonate with respiratory distress. Management in these cases depends on the haemodynamic instability and lung ischemia.

## Background

Neonatal arterial thrombosis is rare with a prevalence of approximately 1 in 40,000 live births and most often occur due to the presence of indwelling catheters [1]. Spontaneous thrombosis of pulmonary artery although rare, has been described in the past [1–5]. Risk factors include septicemia, dehydration, polycythemia, maternal diabetes, asphyxia, and inherited thrombophilias [2, 3]. Most of the neonates with pulmonary artery thrombosis present with features similar to persistent pulmonary hypertension of the newborn (PPHN) or congenital heart diseases [2, 6]. We present a case of term neonate with spontaneous left pulmonary artery (LPA) thrombosis who presented with cyanosis and respiratory distress mimicking PPHN.

## Case presentation

A term female neonate was referred to our centre on day 3 of life in view of respiratory distress with cyanosis. She was born at 40 weeks of gestation by emergency caesarean section due to meconium stained liquor and had smooth perinatal transition with Appearance, Pulse, Grimace, Activity, and Respiration (APGAR) scores of 7 and 9 at 1 and 5 min respectively. However, on examination she was tachypneic with a saturation of 88% in all 4 limbs along with well palpable pulses. She had no murmurs and was hemodynamically stable. Her saturation improved to 96% with supplemental oxygen support with FiO_2_ of 40%. Her chest X-ray was unremarkable and electrocardiogram showed sinus rhythm. Sepsis screen was negative. Both baby and mother were COVID-19 reverse transcription polymerase chain reaction negative.

**Fig. 1:**
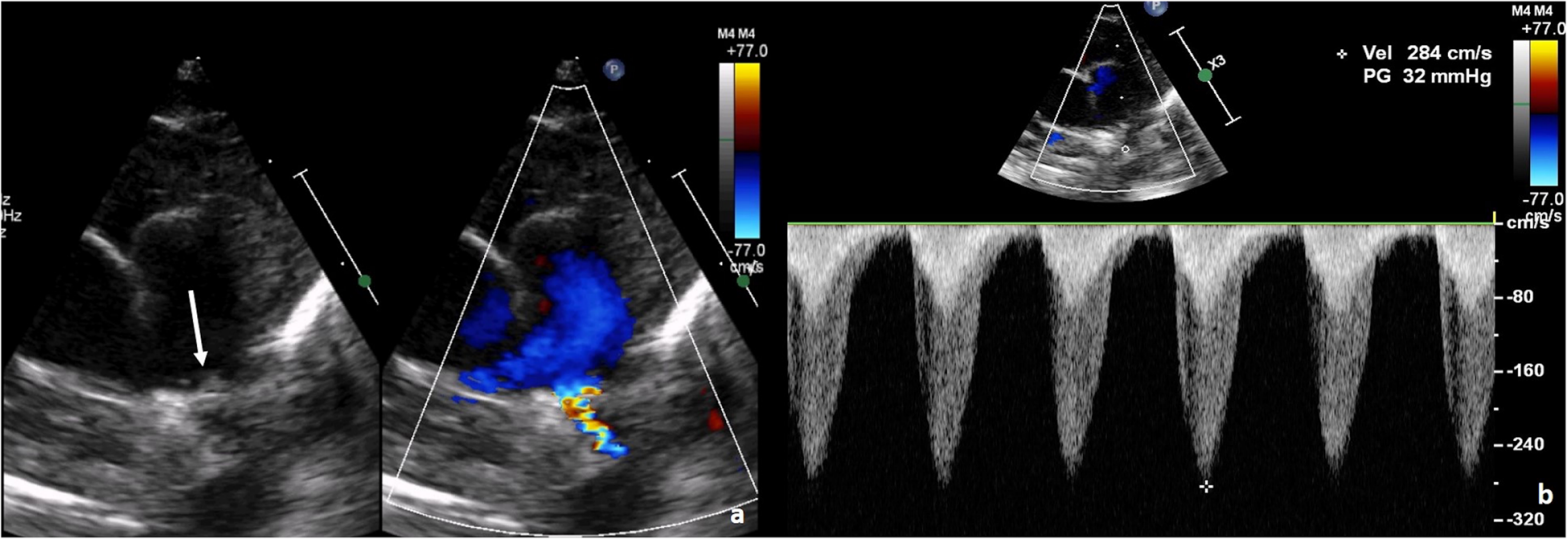
**a** Echocardiogram with colour Doppler in parasternal short axis view showing turbulent flow across LPA along with luminal narrowing (arrow). **b** Echocardiogram with continuous wave Doppler showing gradient across LPA

**Fig. 2:**
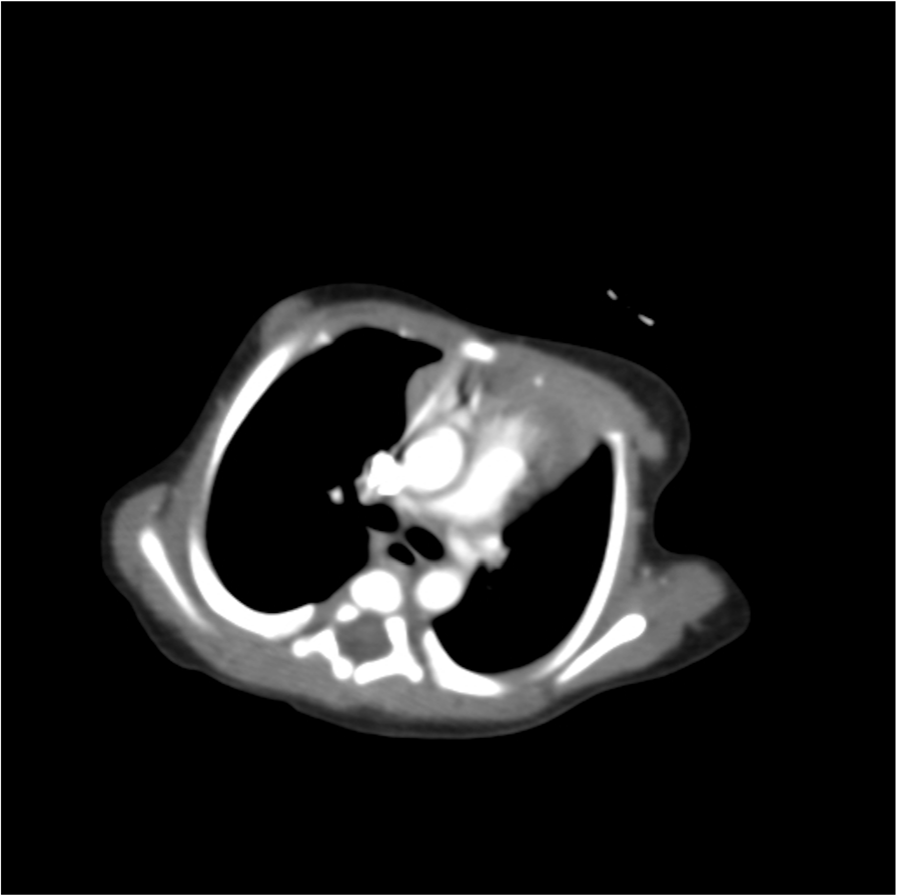
CT pulmonary angiography showing eccentric filling defect at the origin of left pulmonary artery

In view of cyanosis, an echocardiography was done which revealed an inconspicuous LPA. Further focused echocardiogram for LPA showed narrowing at its origin with turbulent flow and a peak gradient of 32 mm of Hg. A bright echogenic thrombus was seen in the LPA extending a tad into main pulmonary artery (MPA) (Figs. 1 and 2). Right pulmonary artery lumen and flow were normal. Computed tomography (CT) chest with pulmonary angiography showed focal eccentric filling defect at the origin of left pulmonary artery suggestive of thrombus and well-developed lung parenchyma. Her D-Dimer levels were elevated (993.89 ng/ml, normal < 240 ng/ml). Her prothrombotic work up including protein C, protein S, Anti thrombin III levels, and Factor V Leiden mutation was negative and parental screen was also unremarkable.

**Fig. 3:**
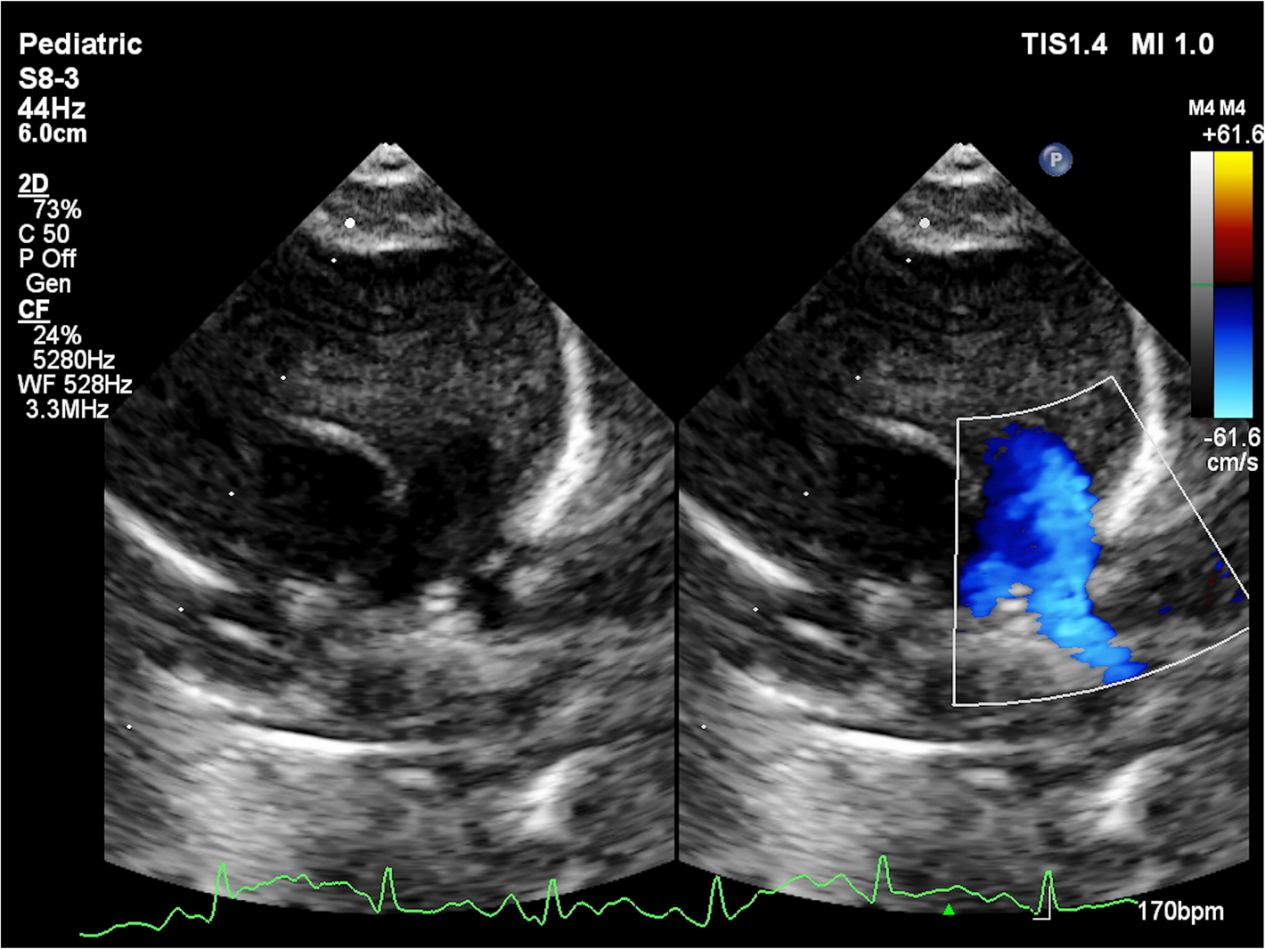
Repeat echocardiogram after 6 weeks of LMWH. Colour Doppler in parasternal short axis view showing partial resolution of thrombus in LPA (arrow)

She was managed with unfractionated heparin infusion (UFH) for 48 h with a target activated partial thromboplastin time (aPTT) of twice the normal. Repeat echocardiogram showed no resolution in thrombus and UFH was changed to LMWH (low molecular weight heparin). Over next 2 weeks with the above management, her tachypnea settled and she was maintaining saturation on room air. She was continued on LMWH for next 6 weeks with 2 weekly echocardiogram to monitor the state of thrombus. Echocardiogram at 6 weeks showed near complete resolution of the thrombus with no gradient across LPA (Fig. 3). LMWH was stopped and she was kept on VKA. Follow-up after 4 weeks of starting VKA showed that the child was doing well with no gradient across the LPA.

## Discussion

We describe a rare case of spontaneous neonatal pulmonary artery thrombosis presenting with respiratory distress and cyanosis. The neonate improved with the use of LMWH. The diagnosis of pulmonary artery thrombosis should be suspected in a neonate without any obvious cause for cyanosis and respiratory distress like in our case. Pulmonary arteries should be assessed carefully during an echocardiographic examination in such cases. This condition can be easily overlooked when there is a partial occlusion of pulmonary arteries and its branches. Even in our case, this condition could be picked up as a result of careful assessment after the inconspicuous LPA drew our attention.

Pulmonary artery thrombosis in neonates is initially suspected based on echocardiogram findings and the diagnosis is established by CT pulmonary angiography (CTPA). CTPA has reduced the need for invasive pulmonary angiography [4]. Further work up has to be focused on identifying all the possible risk factors including prothrombotic states like protein C, protein S, Anti thrombin III levels, Factor V Leiden mutation, sepsis screen, haemoglobin levels for polycythemia, and maternal and child blood sugar levels before labelling pulmonary artery thrombosis as spontaneous [3, 5].

There is no consensus on management of spontaneous pulmonary artery thrombosis and options include anticoagulation, thrombolytic therapy, and mechanical thrombectomy—either surgical or catheter based. Factors which should be considered before choosing a therapeutic modality are hemodynamic instability, lung ischemia, thrombus burden, and risk factors [1, 3–5, 7]. In our case, we managed the with anticoagulation with heparin since the child was haemodynamically stable with no evidence of ischemia. Most of the patients respond to the treatment with better outcomes [1, 3–5].

## Conclusion

Spontaneous pulmonary artery thrombosis is a rare cause of cyanosis and respiratory distress in neonates.

## Data Availability

The datasets used and/or analysed during the current study are available from the corresponding author on reasonable request.

## Abbreviations

PPHN: Persistent pulmonary hypertension of the newborn
CT: Computed tomography
LPA: Left pulmonary artery
CTPA: Computerized tomography pulmonary angiography
LMWH: Low molecular weight heparin
UFH: Unfractionated heparin
aPTT: Activated partial thromboplastin time
MPA: Main pulmonary artery
APGAR: Appearance, pulse, grimace, activity, and respiration
