# Correction to: Spontaneous pulmonary artery thrombus in a neonate

**DOI:** 10.1186/s43044-021-00169-2

**Published:** 2021-05-13

**Authors:** Y. S. Shrimanth, Krishna Prasad, Adari Appala Karthik, Parag Barwad, C. R. Pruthvi, Atit A. Gawalkar, Krishna Santosh, Sanjeev Naganur

**Affiliations:** 1 Department of Cardiology, Advanced Cardiac Centre, Post Graduate Institute for Medical Education and Research (PGIMER) Chandigarh, Sector 12, Chandigarh, 160, 012 India; 2 Department of Pediatrics, Post Graduate Institute for Medical Education and Research (PGIMER) Chandigarh, Chandigarh, India; 3 Department of Cardiology, IMS BHU, Varanasi, India

**Correction to: Egypt Heart J 73, 43 (2021)**

**https://doi.org/10.1186/s43044-021-00167-4**

Following publication of the original article [1], the authors identified an error in the author name of Adari Appala Karthik.

The incorrect author name is: Adari Appala Karhtik

The correct author name is: Adari Appala Karthik

The author group has been updated above and the original article [1] has been corrected.
